# Cultural Engagement Is a Risk-Reducing Factor for Frailty Incidence and Progression

**DOI:** 10.1093/geronb/gbz004

**Published:** 2019-01-08

**Authors:** Nina Trivedy Rogers, Daisy Fancourt

**Affiliations:** 1 Research Department of Epidemiology and Public Health, Institute of Epidemiology and Health Care, University College London, UK; 2 Department of Behavioural Science and Health, Institute of Epidemiology and Health Care, University College London, UK

**Keywords:** Ageing, Cultural engagement, Frailty, Psychosocial

## Abstract

**Objectives:**

Given that frailty is a multifaceted health condition of increasing importance to policy-makers and care providers, it is relevant to consider whether multimodal interventions could provide combined psychophysiological support. As studies have demonstrated the beneficial effects of cultural engagement (including visiting museums/theatre/cinema) for many of the components of frailty, this study sought to explore whether community cultural engagement is associated both with a reduced risk of becoming frail and a slower trajectory of frailty progression in older adults.

**Methods:**

We used data from the English Longitudinal Study of Ageing to measure frequency of cultural engagement and both incident frailty and frailty progression over the following 10 years in 4,575 adults.

**Results:**

Our analyses used competing risks regression models and multilevel growth curve models adjusting for socioeconomic, health behaviors, social confounders, and subthreshold symptoms of frailty. There was a dose–response relationship between increasing frequency of cultural engagement and both incidence and progression of frailty (attendance every few months or more: incidence subhazard ratio = 0.79, 95% confidence interval [CI] = 0.63 to 0.996; trajectory coefficient = –0.0039, 95% CI = –0.0059 to –0.0019).

**Discussion:**

Older adults who engaged in cultural activities every few months or more had a reduced risk of becoming frail and a slower progression of frailty over time. Findings are in line with current calls for multimodal, multifactor, community approaches to support health in older age.

Frailty describes a distinctive age-related health state in which multiple physiological systems within the body decline in function and reserve (Clegg, Young, Iliffe, Rikkert, & Rockwood, 2013). Although related to multi-morbidity and disability (Fried et al., 2004), frailty is distinct in that it encompasses a multitude of health deficits that include but are not limited to mobility, self-reported general health, eyesight, and hearing; being able to carry out basic tasks of daily living; depressive symptoms; and cognitive function (2,3). Frailty is a powerful predictor of functional independence in daily life, care needs, and hospital admissions (Anpalahan & Gibson, 2008; Soong et al., 2015). The high economic, health and social care costs (Comans et al., 2016) associated with frailty are in part drivers of the increased interest in developing cost-effective strategies to reduce the burden of frailty in older adults.

To date, much research on frailty prevention has focused on physical activity (Rogers, Marshall, et al., 2017; Theou et al., 2011). However, given that frailty is a multifaceted health condition that extends more broadly than just functional symptoms, it is relevant to consider whether other multimodal interventions could provide combined psychological and physiological support. Cultural engagement (including going to the theatre, concerts, museums, art galleries, and cinema) is a category of multimodal intervention receiving increasing public health interest (12). Cultural engagement has psychological, social, and behavioral components that in themselves are all known to be protective for frailty. For example, cultural engagement is cognitively stimulating and supports the development of cognitive reserve (Fancourt & Steptoe, 2018b; Fancourt, Steptoe, & Cadar, 2018). It also supports emotion regulation, stress reduction, wellbeing and helps protect against depression (Fancourt & Steptoe, 2018a; Fancourt & Tymoszuk, 2018). It is social so reduces loneliness (Todd, Camic, Lockyer, Thomson, & Chatterjee, 2017). It is also a form of mild-moderate activity so reduces sedentary behaviors, supports balance and gait, and is associated with a lower risk of chronic pain in older age (Fancourt & Steptoe, 2018c). However, to date, there have been no research studies specifically looking at the relationship between cultural engagement and frailty, and the frequency of engagement required remains unknown. Consequently, this study sought to explore whether community cultural engagement is associated both with a reduced risk of developing frailty and a slower trajectory of frailty progression in older adults.

## Method

### Participants

We used data from the English Longitudinal Study of Ageing (ELSA), a longitudinal cohort study that contains a representative sample of adults aged more than 50 years living in England (18). We specifically worked with data from wave 2 (2004/2005) across every biennial wave through to wave 7 (2014/2015); a total of six waves and a decade of data. Of the eligible 9,432 participants at baseline, we excluded 3,973 for missing data on exposure or covariates at baseline and excluded a further 1,144 who were already classified as frail at baseline, providing an analytic sample of 4,575 (see Supplementary material for a diagram of participants included). The study received ethical approval from the National Research Ethics Service and all participants provided informed consent.

### Measures

We measured cultural engagement using participant self-reports at wave 2 of frequency of visits to (a) the theatre, concert, or opera; (b) the cinema; and (c) an art gallery, exhibition, or museum. We combined responses from these three variables to create an overall frequency of cultural engagement, with responses coded as never, less than once a year, once or twice a year, and every few months or more (Supplementary Table 1).

Frailty was measured biennially over a duration of 10 years (waves 2–7). Using criteria set out by Rockwood and colleagues (Searle, Mitnitski, Gahbauer, Gill, & Rockwood, 2008), we used a 56-item frailty index (FI) that comprises variables for chronic conditions, eyesight, hearing, general health, disability, mobility, depression, and cognitive function (Rogers, Marshall, et al., 2017; Rogers, Steptoe, & Cadar, 2017). The standard FI threshold to denote frailty is a score of .25 or higher (range 0–1), and this is equivalent to the expression of at least 14 of the 56 available frailty indicators that comprise the FI (Rockwood, Andrew, & Mitnitski, 2007). Further details of the composition of the FI are provided in Supplementary Table 2.

Covariates used in the analysis were selected based on previous studies using the same FI (Rogers, Marshall, et al., 2017; Rogers et al., 2017): age (in years), gender, educational qualifications (no educational qualifications vs one or more qualification), non-pension wealth quintile (poorest vs higher) (22), living status (alone vs with a partner), level of physical activity (sedentary/low vs moderate/vigorous weekly activity), organizational membership (whether a member of an organization, group, or club or not), and number of social engagements per week (including meeting, telephoning, or emailing family and friends).

### Statistical Analysis

The association between frequency of engaging in cultural activities and incident frailty (FI > .25) was examined using competing risks regression models and according to the methodology of Fine and Gray (1999). Cumulative incidence function models were produced to predict the risk of participants becoming frail in relation to their level of community cultural engagement while taking into account death as a competing event. Participant age was used as the time variable in the survival model. The event of interest was the date when a sufficient proportion of health deficits were reported and participants were defined as frail. Date of death was recorded as the competing-risk event and the study was censored at the last date of contact or February 1, 2013, which marked the end of the study. Month and year of death, up until February 1, 2013 was used for those participants who had given written consent for linkage to official records from the National Health Service central register. The assumption of proportional hazards distribution for each covariate was tested using the Scheike and Zhang (2008) test and was not violated (*p* values ≥ .05).

To calculate the trajectory of frailty progression in older adults, multilevel growth curve models were used to approximate the rate of progression of FI scores between waves 2 and 7 of ELSA and according to frequency of engagement in cultural activities. Negative β coefficients indicate a slower rate of progression of frailty compared with the reference group (never engaging in cultural activities).

**Figure 1.**
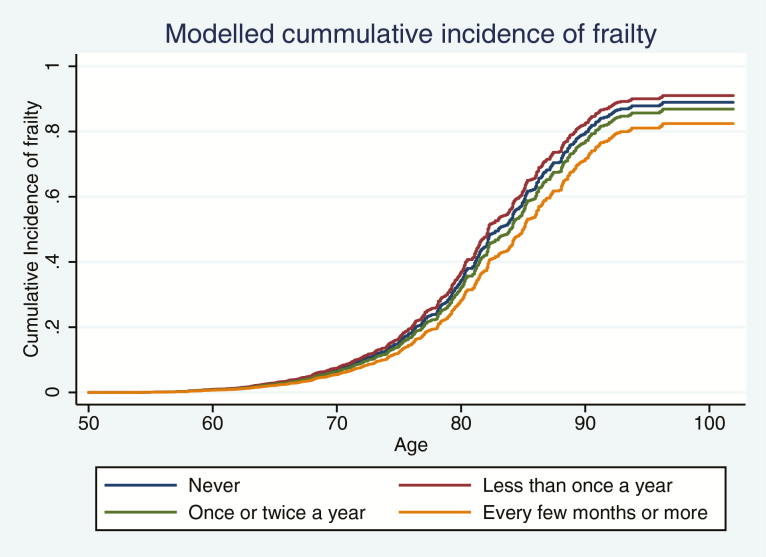
Modeled cumulative incidence of frailty by frequency of cultural engagement, adjusted for all covariates and accounting for the competing risk of death.

**Figure 2.**
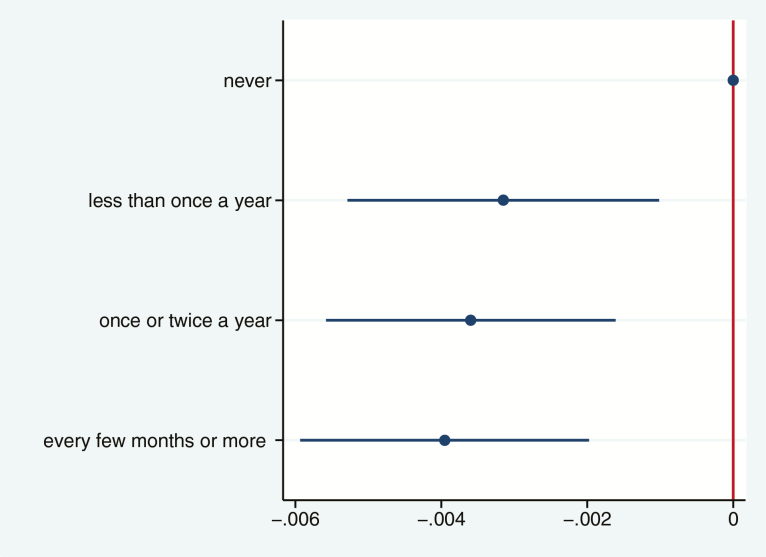
Parameter estimates and 95% confidence intervals depicting the average 10-year frailty trajectories predicted by baseline frequency of cultural engagement.

For both incidence and trajectory, Model 1 was adjusted for demographic, behavioral and social covariates. Following the statistical procedures of previous studies using frailty indices (Song, Mitnitski, & Rockwood, 2010), Model 2 additionally adjusted for health-related confounders (including chronic condition, poor eyesight or hearing, poor general health, disability, mobility problems, depression, and/or declining cognitive function). This adjustment was made with a binary variable of whether participants were non-frail (score 0–.08: exhibiting very few health-related problems) or pre-frail (score .08–.25: exhibiting several health-related problems). Adjusted and unadjusted data for both models are shown in Table 1 and fully adjusted models are shown in Figures 1 and 2.

**Table 1.** Adjusted and Unadjusted Models Showing Associations Between Cultural Engagement and Frailty Incidence and Trajectory

Frailty incidence				
	Model 1: Adjusted for covariates			
	SHR	*SE*	*p*	CI
Never	REF			
Less than once a year	1.04	0.12	.72	0.83 to 1.31
Once or twice a year	0.84	0.09	.12	0.68 to 1.04
Every few months or more	**0.72**	**0.08**	**.004**	**0.57 to 0.90**
	Model 2: Adjusted for covariates and baseline frailty (non-frail vs pre-frail)			
	SHR	*SE*	*p*	CI
Never	REF			
Less than once a year	1.09	0.13	.45	0.87 to 1.38
Once or twice a year	0.92	0.10	.46	0.74 to 1.14
Every few months or more	**0.79**	**0.09**	**.047**	**0.63 to 0.996**
Frailty trajectory				
	Model 1: Adjusted for covariates			
	Coef	*SE*	*p*	CI
Never	REF			
Less than once a year	**–0.0029**	**0.001**	**.006**	**–0.0050 to –0.0008**
Once or twice a year	**–0.0032**	**0.001**	**.002**	**–0.0051 to –0.0012**
Every few months or more	**–0.0035**	**0.001**	**<.001**	**–0.0054 to –0.0015**
	Model 2: Adjusted for covariates and baseline frailty (non-frail vs pre-frail)			
	Coef	*SE*	*p*	CI
Never	REF			
Less than once a year	**–0.0031**	**0.001**	**.004**	**–0.0053 to -–0.0010**
Once or twice a year	**–0.0035**	**0.001**	**.001**	**–0.0055 to –0.0015**
Every few months or more	**–0.0039**	**0.001**	**<.001**	**–0.0059 to –0.0019**

*Note*. Bold values indicate significance at p < .05; CI = 95% confidence intervals; Coef = coefficient; SHR = subhazard ratio; SE = standard error.

We tested our assumptions in a series of sensitivity analyses (all shown in Supplementary Tables). First, because a number of participants were missing data on covariates, we also ran minimally adjusted models (age and sex only), which allowed us a larger sample size. To assess whether subthreshold symptoms of frailty might have affected cultural engagement and predisposed people to becoming frail, we restricted the sample to exclude participants who became frail at wave 3 and also excluded those in the top 10% of subthreshold frailty scores at baseline. Second, although it has previously been shown that patterns of cultural engagement are relatively stable in older age (DCMS, 2017), we tested potential changes in individual patterns of cultural engagement by rerunning our analyses using an averaged frequency of cultural engagement over waves 2 and 3. Specifically for trajectory analyses, we ran two further sensitivity analyses. To assess whether cultural engagement is also associated with a reduced trajectory of frailty in people with frailty, we reran the analyses including both frail and non-frail participants. Finally, although adjusting for specific baseline frailty score is not recommended when undertaking analyses of *incidence* (Song et al., 2010), we did run analyses on frailty *trajectory* including specific baseline frailty. All analyses were conducted using Stata 14 (StataCorp LP, College Station, TX).

## Results

### Demographics

Participants had a mean age of 64.7 years (*SD* = 8.8, range = 52–99) and were 52.7% female. Of them, 15.7% participants never engaged in cultural activities, 16.4% engaged less than once a year, 26.5% engaged once or twice a year, and 41.3% engaged every few months or more. Full demographics are provided in Supplementary Material. Cultural engagement showed a moderate association with broader social engagement (*r* = .43, *p* < .001) and a small negative association with broader civic engagement (*r* = –.26, *p* < .001).

### Frailty Incidence

There was a dose–response relationship between frequency of cultural engagement and incident frailty (trend: subhazard ratio = 0.92, 95% confidence interval [CI] = 0.85 to 0.98; Figure 1 and Table 1). Compared with participants who never engaged in cultural activities, participants who engaged in cultural activities every few months or more were less likely to become frail over time. These results were robust after adjusting for socioeconomic and behavioral risk factors and baseline frailty.

### Frailty Progression

Frequency of engagement in cultural activities at baseline was also associated with a slower trajectory of frailty (Figure 2). There was evidence of a dose–response relationship with any level of engagement associated with a slower rate of frailty progression (trend: coefficient = –0.0010, 95% CI = –0.0016 to –0.0004; Table 1). Participants had a 1.3 times increased risk of becoming frail by the age of 80 if they did not engage in cultural activities compared with if they engaged at least every few months or more. These findings were robust to the inclusion of all covariates.

### Sensitivity Analyses

Sensitivity analyses for frailty incidence and trajectories confirmed that results were similar when using a larger sample size and minimally adjusted models (Supplementary Tables 3 and 7) and were unaffected by the use of averaged cultural behavior across two waves rather than cultural behavior just at a single time-point (Supplementary Tables 6 and 10). Excluding participants who became frail at wave 3 did not affect frailty trajectories (Supplementary Table 8) but did reduce the number of incident frailty cases by 32%, leading to an attenuation of results and a loss of significance (Supplementary Table 4). However, when using an alternative approach and excluding the top 10% of subthreshold frailty scores at baseline, all results for incidence and trajectories were maintained (Supplementary Tables 5 and 9). Finally, additionally including participants who were already categorized as frail at baseline and adjusting for baseline frailty as a continuous variable did not alter the significance of the results (Supplementary Tables 11 and 12).

## Discussion

This study showed for the first time that cultural engagement in older age is associated with both a reduced risk of incident frailty and a slower trajectory of frailty progression in older adults. These results were found independent of demographic, socioeconomic, and social confounders. They were also found independent of whether participants showed signs of being “pre-frail” at baseline, indicative of participants having multiple health-impairing factors such as a chronic condition, poor eyesight or hearing, poor general health, disability, mobility problems, depression, and/or declining cognitive function. Results for frailty trajectory were particularly robust, showing no signs of attenuation across any sensitivity analyses. Results for frailty incidence were maintained when considering possible changing patterns of cultural behavior but attenuated when excluding participants who became frail at wave 3. However, this reduced the number of frailty cases so affected statistical power within the analyses.

Our results support findings from a previous cross-sectional study, showing associations between cultural engagement and a lower probability of being frail (Poli et al., 2017). However, we were able to extend these previous findings by exploring longitudinal associations. In theorizing why we found these results, previous studies have suggested that the health benefits of cultural engagement could be due to its combined effect on reducing social isolation, sedentary behaviors, and stress while providing social support networks and cognitive stimulation (Camic & Chatterjee, 2013). However, analyses have repeatedly found health-related benefits of cultural engagement even when controlling for these broader states (Fancourt & Steptoe, 2018c, 2018a, 2018b; Fancourt & Tymoszuk, 2018), suggesting that other aspects of cultural engagement such as hedonic experiences, emotional expression, and providing a sense of meaning and purpose may also be key (Steptoe & Fancourt, 2019).

Strengths of this study include the use of a large, prospective and representative analytical sample and long-term follow-up of participants. The study also benefited from a validated measure of frailty. However, it is still possible that results are bi-directional and may have been affected by reverse causality because being frailer might be associated with reduced likelihood of engaging in cultural activities. Nevertheless, the sample was restricted to include only non-frail adults at baseline and we included subthreshold symptoms of frailty in our analyses, which did not affect our significant results. Further, results for frailty trajectories were maintained when excluding participants who became frail shortly after baseline (who therefore may have been exhibiting impaired behaviors already) and also when adjusting stringently for precise baseline frailty score. The study was also limited by self-reported cultural engagement and covariates. Further, we acknowledge that frailty has competing definitions. In this study, we used the FI approach, considering accumulated health deficits, but our results may differ from those studies using different definitions or indices (Mudge & Hubbard, 2018).

The findings here of the benefits of cultural engagement are in line with current calls for multimodal, multifactor, community approaches to support health in older age (26). Given that frailty is associated with falls, delirium, fluctuating disability, increased care needs and increased use of health services, the identification that engagement with existing community cultural activities is protective is important. Although our analyses found that 83% of older adults engage in cultural activities, only 41.3% do so at the level required for benefits on frailty incidence or trajectory to be felt. Notably, while many health interventions involve the large-scale implementation of new interventions, cultural venues already exist, with over 40,000 museums across Europe, the United States, and Canada alone (Camic & Chatterjee, 2013). These sites are arguably underutilized within public health at present yet offer strong potential for supporting health in older adults, largely because as well as combining multiple health-promoting factors, their aesthetic content provides an intrinsic motivation to engage. It is therefore recommended that future behavior change intervention studies are designed focused on increasing cultural engagement in older adults, especially those at risk of becoming frail.

## Supplementary Material

gbz004_suppl_Supplementary_Material
