# Supplementary material for: Cultural Engagement Is a Risk-Reducing Factor for Frailty Incidence and Progression
Source: J Gerontol B Psychol Sci Soc Sci. 2019 Jan 8;75(3):571–6. doi: 10.1093/geronb/gbz004 (PMC7768715; doi:10.1093/geronb/gbz004)
Supplement: gbz004_suppl_Supplementary_Material [file gbz004_suppl_supplementary_material.docx]

**Cultural engagement is a risk-reducing factor for frailty incidence and progression in non-frail adults**

Supplementary Materials

Rogers, N.T & Fancourt D

Methods

**Demographics**

Sample characteristics were created according to frequency of cultural engagement. Means and standard deviations were used to describe variables with continuous data and numbers and percentages were used for variables that were defined using categorical data. The study’s aim was to determine whether the frequency with which participants engage in cultural activities is associated with trajectories and the development of frailty up to 10 years later. Participants were excluded from the study if they had insufficient variables to derive a FI; or missing data on cultural engagement and baseline covariates or if they were frail (FI≥ 0.25) at wave 2.

**Construction of the Frailty Index**

To compute the FI, variables included were assigned a score of 0-1: a value of 1 was assigned for each deficit that was present and a value of 0 was assigned for every deficit that was absent. A score between 0 and 1 was used for some variables when a participant indicated there was not full but partial expression of a health deficit. The FI was created by adding up the total number of health deficits and dividing by the number of deficits being considered. At least 30 frailty variables were required to derive a FI (Rockwood & Mitnitski, 2007). The analytical sample comprised of adults who were non-frail (FI < 0.25) at baseline (Rockwood, Andrew, & Mitnitski, 2007). All had sufficient health variables to create a FI comprised of at least 30 variables. A total of 4380 participants (95.7%) had non-missing data for all 56 variables, 166 (3.63%) had non-missing data for 55 variables, and the remaining 29 (0.6%) participants had non-missing data for between 42–54 variables.

**Statistics**

Incidence: A total of 831 participants developed frailty in the follow-up period and 289 died.

Trajectory: There were a total of 23,436 observations and participants provided an average of 5.1 observations across the 6 time-points.

**References**

Rockwood, K., Andrew, M., & Mitnitski, A. (2007). A comparison of two approaches to measuring frailty in elderly people. *The Journals of Gerontology. Series A, Biological Sciences and Medical Sciences*, *62*(7), 738–743.

Rockwood, K., & Mitnitski, A. (2007). Frailty in relation to the accumulation of deficits. *The Journals of Gerontology. Series A, Biological Sciences and Medical Sciences*, *62*(7), 722–727.

Participants

**Supplementary Figure 1: Inclusion of participants in study analysis**

Participants that were frail at baseline

N= 1144

Missing covariates at baseline

N = 1127

Core participants with no missing exposure **N=6846**

Non-frail core participants with no missing exposure, or outcome variables in analytical sample **N=4575**

Core participants at baseline (wave 2) **N=9432**

Missing cultural engagement

N = 2846

Demographics

**Supplementary Table 1: Demographic characteristics of the non-frail participants of the analytical sample by frequency of cultural engagement (n=4575).**

|  | Total population | Never | Less than once a year | Once/twice a year | Every few months or more |
| --- | --- | --- | --- | --- | --- |
| Total sample N (%) | 4575 (100) | 722 (15.8) | 750 (16.4) | 1,213 (26.5) | 1,890 (41.3) |
| Frailty Index score -Mean (SD) | 0.11 (0.06) | 0.13 (0.06) | 0.11 (0.06) | 0.10 (0.05) | 0.09 (0.05) |
| Age - Mean (SD) | 64.7 (8.80) | 68.4 (9.68) | 64.7 (8.85) | 64.5 (8.57) | 63.3 (8.15) |
| Female N (%) | 2409 (52.7) | 353 (48.9) | 363 (48.4) | 624 (51.4) | 1069 (56.6) |
| Poorest wealth quintile N (%) | 489 (10.7) | 199 (27.6) | 91 (12.1) | 104 (8.57) | 95(5.03) |
| No educational qualifications N (%) | 1280 (28.0) | 427 (59.1) | 256 (34.1) | 316 (26.1) | 281 (14.9) |
| Lives alone N (%) | 1186 (25.9) | 255 (35.3) | 180 (24.0) | 289 (23.8) | 462 (24.4) |
| No of social engagements /week Mean (SD) | 4.70 (1.85) | 3.95 (1.81) | 4.51 (1.81) | 4.67 (1.81) | 5.07(1.80) |
| Not a member of an organisation, club or society N (%) | 997(21.8) | 297 (41.1) | 213 (28.4) | 242 (20.0) | 245 (13.0) |
| Sedentary/low physical activity N (%) | 592 (12.9) | 204 (28.3) | 95(12.7) | 144 (11.9) | 149 (7.88) |

Frailty

**Supplementary Table 2: Deficits included in the 56-item frailty index and their corresponding values**

| Domain | Frailty components |  |
| --- | --- | --- |
| Mobility Difficulties | Walking 100 yards | Yes = 1; No = 0 |
|  | Sitting for about two hours | Yes = 1; No = 0 |
|  | Getting up from a chair after sitting for long periods | Yes = 1; No = 0 |
|  | Climbing several flights of stairs without resting | Yes = 1; No = 0 |
|  | Climbing one flight of stairs without resting | Yes = 1; No = 0 |
|  | Stooping, kneeling, or crouching | Yes = 1; No = 0 |
|  | Reaching or extending arms above shoulder level | Yes = 1; No = 0 |
|  | Pulling or pushing large objects like a living room chair | Yes = 1; No = 0 |
|  | Lifting or carrying weights over 10 pounds, like a heavy bag | Yes = 1; No = 0 |
|  | Picking up a 5p coin from a table | Yes = 1; No = 0 |
| Disability (ADL/iADL) | Dressing, including putting on shoes and socks | Yes = 1; No = 0 |
|  | Walking across a room | Yes = 1; No = 0 |
|  | Bathing or showering | Yes = 1; No = 0 |
|  | Eating, such as cutting up your food | Yes = 1; No = 0 |
|  | Getting in or out of bed | Yes = 1; No = 0 |
|  | Using the toilet, including getting up or down | Yes = 1; No = 0 |
|  | Using a map to figure out how to get around in a strange place | Yes = 1; No = 0 |
|  | Preparing a hot meal | Yes = 1; No = 0 |
|  | Shopping for groceries | Yes = 1; No = 0 |
|  | Making telephone calls | Yes = 1; No = 0 |
|  | Taking medication | Yes = 1; No = 0 |
|  | Managing money, (e.g. paying bills and keeping track of expenses) | Yes = 1; No = 0 |
|  | Doing work around the house or garden | Yes = 1; No = 0 |
| General health | Self-reported general health | Excellent = 0; Very good = 0.25; Good = 0.5; Fair = 0.75; Poor = 1 |
| Depressive Symptoms | Respondent felt depressed much of the time during past week | Yes = 1; No = 0 |
|  | Respondent felt that everything they did during the past week was an effort | Yes = 1; No = 0 |
|  | Respondent felt that their sleep was restless during the past week | Yes = 1; No = 0 |
|  | Respondent was not happy much of the time during the past week | Yes = 1; No = 0 |
|  | Respondent felt lonely much of the time during the past week | Yes = 1; No = 0 |
|  | Respondent did not enjoy life much of the time during the past week | Yes = 1; No = 0 |
|  | Respondent felt sad much of the time during the past week | Yes = 1; No = 0 |
|  | Respondent could not get going much of the time during the past week | Yes = 1; No = 0 |
| Self-reported conditions | High blood pressure or hypertension | Yes = 1; No = 0 |
|  | Angina | Yes = 1; No = 0 |
|  | Heart attack (including myocardial infarction or coronary thrombosis) | Yes = 1; No = 0 |
|  | Congestive heart failure | Yes = 1; No = 0 |
|  | An abnormal heart rhythm | Yes = 1; No = 0 |
|  | Diabetes or high blood sugar | Yes = 1; No = 0 |
|  | A stroke (cerebral vascular disease) | Yes = 1; No = 0 |
|  | Chronic lung disease such as chronic bronchitis or emphysema | Yes = 1; No = 0 |
|  | Asthma | Yes = 1; No = 0 |
|  | Arthritis (including osteoarthritis, or rheumatism) | Yes = 1; No = 0 |
|  | Osteoporosis, sometimes called thin or brittle bones | Yes = 1; No = 0 |
|  | Cancer or a malignant tumour (excluding minor skin cancers) | Yes = 1; No = 0 |
|  | Parkinson's disease | Yes = 1; No = 0 |
|  | Any emotional, nervous or psychiatric problems | Yes = 1; No = 0 |
|  | Alzheimer's disease | Yes = 1; No = 0 |
|  | Dementia, organic brain syndrome, senility or other serious memory impairment | Yes = 1; No = 0 |
|  | Eyesight (while using lenses, if appropriate) poor compared to excellent | Excellent = 0; Very good = 0.2; Good = 0.4; Fair = 0.6; Poor = 0.8; Blind = 1 |
|  | Hearing (while using hearing aid, if appropriate) poor compared to excellent | Excellent = 0; Very good = 0.25; Good = 0.5; Fair = 0.75; Poor = 1 |
| Cognitive function | Successfully Identified today's date | Yes = 1; No = 0 |
|  | Successfully Identified today's month | Yes = 1; No = 0 |
|  | Successfully Identified today's year | Yes = 1; No = 0 |
|  | Successfully Identified the day of the week | Yes = 1; No = 0 |
|  | Immediate word recall (lowest quintile) | 1^st^ Quintile = 0; 2^nd^ Quintile = 0.25; 3^rd^ Quintile = 0.5; 4^th^ Quintile = 0.75; 5^th^ Quintile = 1 |
|  | Delayed word recall (lowest quintile) | 1^st^ Quintile = 0; 2^nd^ Quintile = 0.25; 3^rd^ Quintile = 0.5; 4^th^ Quintile = 0.75; 5^th^ Quintile = 1 |

Frailty incidence

**Supplementary Table 3: frailty incidence with only minimal adjustment (preserving a larger sample size due to inclusion of participants with missing covariates)**

|  | Adjusted for age and sex only | | | |
| --- | --- | --- | --- | --- |
|  | SHR | SE | p | CI |
| Never | REF |  |  |  |
| Less than once a year | **0.80** | **0.08** | **.024** | **0.66-0.97** |
| Once or twice a year | **0.62** | **0.06** | **<.001** | **0.52-0.74** |
| Every few months or more | **0.48** | **0.04** | **<.001** | **0.41-0.57** |

SHR: subhazard ratio; SE: standard error; CI: 95% confidence intervals

N=5,239, Number of frailty cases=1,015

**Supplementary Table 4: frailty incidence excluding participants who became frail at wave 3**

|  | Model 1: Adjusted for covariates | | | |
| --- | --- | --- | --- | --- |
|  | SHR | SE | p | CI |
| Never | REF |  |  |  |
| Less than once a year | 1.07 | 0.15 | .63 | 0.81-1.42 |
| Once or twice a year | 0.91 | 0.12 | .49 | 0.70-1.19 |
| Every few months or more | 0.76 | 0.11 | .052 | 0.58-1.00 |
|  | Model 2: Adjusted for covariates  and baseline frailty (non-frail vs pre-frail) | | | |
|  | SHR | SE | p | CI |
| Never | REF |  |  |  |
| Less than once a year | 1.13 | 0.17 | .43 | 0.84-1.50 |
| Once or twice a year | 1.00 | 0.14 | >.99 | 0.77-1.30 |
| Every few months or more | 0.83 | 0.12 | .20 | 0.63-1.10 |

SHR: subhazard ratio; SE: standard error; CI: 95% confidence intervals

Number of frailty cases=561

**Supplementary Table 5: frailty incidence excluding those in the top 10% of frailty at baseline**

|  | Adjusted for covariates  and excluding those in top 10% of baseline frailty | | | |
| --- | --- | --- | --- | --- |
|  | SHR | SE | p | CI |
| Never | REF |  |  |  |
| Less than once a year | 1.04 | 0.12 | .77 | 0.82-1.31 |
| Once or twice a year | 0.85 | 0.10 | .15 | 0.68-1.06 |
| Every few months or more | **0.74** | **0.09** | **.01** | **0.59-0.93** |

SHR: subhazard ratio; SE: standard error; CI: 95% confidence intervals

N=4,109, Number of frailty cases=829

**Supplementary Table 6: frailty incidence using an average of cultural engagement across waves 2 & 3**

|  | Model 1: Adjusted for covariates | | | |
| --- | --- | --- | --- | --- |
|  | SHR | SE | p | CI |
| Never | REF |  |  |  |
| Less than once a year | 1.05 | 0.12 | .66 | 0.84-1.33 |
| Once or twice a year | 0.94 | 0.11 | .57 | 0.75-1.17 |
| Every few months or more | **0.70** | **0.08** | **.003** | **0.55-0.89** |
|  | Model 2: Adjusted for covariates  and baseline frailty (non-frail vs pre-frail) | | | |
|  | SHR | SE | p | CI |
| Never | REF |  |  |  |
| Less than once a year | 1.11 | 0.13 | .36 | 0.88-1.41 |
| Once or twice a year | 0.99 | 0.11 | .93 | 0.79-1.24 |
| Every few months or more | **0.75** | **0.09** | **.02** | **0.59-0.96** |

SHR: subhazard ratio; SE: standard error; CI: 95% confidence intervals

Frailty trajectory

**Supplementary Table 7: frailty trajectory with only minimal adjustment (preserving a larger sample size due to inclusion of participants with missing covariates)**

|  | Adjusted for age and sex only | | | |
| --- | --- | --- | --- | --- |
|  | Coef | SE | p | CI |
| Never | REF |  |  |  |
| Less than once a year | **-0.0036** | **0.001** | **<.001** | **-0.0054 to -0.0017** |
| Once or twice a year | **-0.0044** | **0.001** | **<.001** | **-0.0060 to -0.0027** |
| Every few months or more | **-0.0051** | **0.001** | **<.001** | **-0.0066 to -0.0036** |

Coef: coefficient; SE: standard error; CI: 95% confidence intervals

**Supplementary Table 8: frailty trajectory excluding participants who became frail at wave 3**

|  | Model 1: Adjusted for covariates | | | |
| --- | --- | --- | --- | --- |
|  | Coef | SE | p | CI |
| Never | REF |  |  |  |
| Less than once a year | **-0.0029** | **0.001** | **.007** | **-0.0049 to -0.0008** |
| Once or twice a year | **-0.0032** | **0.001** | **.001** | **-0.0051 to -0.0012** |
| Every few months or more | **-0.0034** | **0.001** | **.001** | **-0.0053 to -0.0014** |
|  | Model 2: Adjusted for covariates  and baseline frailty (non-frail vs pre-frail) | | | |
|  | Coef | SE | p | CI |
| Never | REF |  |  |  |
| Less than once a year | **-0.0031** | **0.001** | **.004** | **-0.0052 to -0.0010** |
| Once or twice a year | **-0.0035** | **0.001** | **<.001** | **-0.0055 to -0.0016** |
| Every few months or more | **-0.0038** | **0.001** | **<.001** | **-0.0058 to -0.0019** |

Coef: coefficient; SE: standard error; CI: 95% confidence intervals

N=4,304

**Supplementary Table 9: frailty trajectory excluding those in the top 10% of frailty at baseline**

|  | Adjusted for covariates  and excluding those in top 10% of baseline frailty | | | |
| --- | --- | --- | --- | --- |
|  | Coef | SE | p | CI |
| Never | REF |  |  |  |
| Less than once a year | **-0.0069** | **0.001** | **<.001** | **-0.0093 to -0.0045** |
| Once or twice a year | **-0.0076** | **0.001** | **<.001** | **-0.0098 to -0.0055** |
| Every few months or more | **-0.0093** | **0.001** | **<.001** | **-0.0113 to -0.0073** |

Coef: coefficient; SE: standard error; CI: 95% confidence intervals

**Supplementary Table 10: frailty trajectory using an average of cultural engagement across waves 2 & 3**

|  | Model 1: Adjusted for covariates | | | |
| --- | --- | --- | --- | --- |
|  | Coef | SE | p | CI |
| Never | REF |  |  |  |
| Less than once a year | **-0.0024** | **0.001** | **.039** | **-0.0046 to -0.0001** |
| Once or twice a year | **-0.0030** | **0.001** | **.007** | **-0.0051 to -0.0008** |
| Every few months or more | **-0.0038** | **0.001** | **<.001** | **-0.0060 to -0.0017** |
|  | Model 2: Adjusted for covariates  and baseline frailty (non-frail vs pre-frail) | | | |
|  | Coef | SE | p | CI |
| Never | REF |  |  |  |
| Less than once a year | **-0.0026** | **0.001** | **.025** | **-0.0049 to -0.0003** |
| Once or twice a year | **-0.0034** | **0.001** | **.002** | **-0.0055 to -0.0012** |
| Every few months or more | **-0.0044** | **0.001** | **<.001** | **-0.0066 to -0.0023** |

Coef: coefficient; SE: standard error; CI: 95% confidence intervals

**Supplementary Table 11: frailty trajectory including participants with frailty at baseline**

|  | Model 1: Adjusted for covariates | | | |
| --- | --- | --- | --- | --- |
|  | Coef | SE | p | CI |
| Never | REF |  |  |  |
| Less than once a year | -0.0018 | 0.001 | .078 | -0.0038 to 0.0002 |
| Once or twice a year | -0.0015 | 0.001 | .11 | -0.0034 to 0.0004 |
| Every few months or more | -0.0014 | 0.001 | .14 | -0.0033 to 0.0004 |
|  | Model 2: Adjusted for covariates  and baseline frailty (non-frail vs pre-frail) | | | |
|  | Coef | SE | p | CI |
| Never | REF |  |  |  |
| Less than once a year | **-0.0026** | **0.001** | **.01** | **-0.0046 to -0.0006** |
| Once or twice a year | **-0.0028** | **0.001** | **.003** | **-0.0047 to -0.0009** |
| Every few months or more | **-0.0029** | **0.001** | **.003** | **-0.0048 to -0.0010** |

Coef: coefficient; SE: standard error; CI: 95% confidence intervals

**Supplementary Table 12: frailty trajectory adjusting for baseline frailty as a continuous variable**

|  | Model 2: Adjusted for covariates  and baseline frailty (non-frail vs pre-frail) | | | |
| --- | --- | --- | --- | --- |
|  | Coef | SE | p | CI |
| Never | REF |  |  |  |
| Less than once a year | **-0.0032** | **0.001** | **.005** | **-0.0055 to -0.0010** |
| Once or twice a year | **-0.0037** | **0.001** | **.001** | **-0.0058 to -0.0016** |
| Every few months or more | **-0.0042** | **0.001** | **<.001** | **-0.0063 to -0.0021** |

Coef: coefficient; SE: standard error; CI: 95% confidence intervals
